# Effect of Birdsong Soundscape on Perceived Restorativeness in an Urban Park

**DOI:** 10.3390/ijerph17165659

**Published:** 2020-08-05

**Authors:** Wei Zhao, Hongyu Li, Xun Zhu, Tianji Ge

**Affiliations:** 1 School of Architecture, Harbin Institute of Technology, Key Laboratory of Cold Region Urban and Rural Human Settlement Environment Science and Technology, Ministry of Industry and Information Technology, Harbin 150006, China; zhaoweila@hit.edu.cn (W.Z.); 19S034112@stu.hit.edu.cn (H.L.); getj@vanke.com (T.G.); 2 China Vanke Co., Ltd., Vanke Center 33 Huanmei Road Dameisha Yantian District, Shenzhen 518083, China

**Keywords:** soundscape, birdsong, perceived restorativeness, urban park

## Abstract

Natural soundscapes have beneficial effects on the perceived restorativeness of an environment. This study examines the effect of birdsong, a common natural soundscape, on perceived restorativeness in Harbin Sun Island Park in China. Eight sites were selected and a series of questionnaire surveys on perceived restorativeness soundscape scale (PRSS) of four birdsong types were conducted during summer and winter. Two-hundred and forty respondents participated in this survey. Analysis of the survey results shows that different types of birdsong have different perceived restorativeness effects in different seasons. Crow birdsong has the worst effect on the perceived restorativeness in both summer and winter. Moreover, sound comfort and preference are significantly associated with the perceived restorativeness. The perceived restorativeness soundscape is best when birdsong is at a height of 4 m rather than 0.5 m or 2 m. The demographic/social factors of age, education, and stress level are all correlated with perceived restorativeness. There are suggestions for urban park design, especially with constructed natural elements. Creating a suitable habitat for multiple species of birds will improve perceived restorativeness. Moreover, appropriate activities should be provided in city parks to ensure restorativeness environments, especially for subjects with high levels of education and stress.

## 1. Introduction

Noise is an important public health issue and acoustic environment is an important factor in creating sustainable and healthy cities. Exposure to noise can lead to auditory and non-auditory effects on human health, especially following long-term exposure [1]. Noise-induced hearing loss remains highly prevalent in occupational settings and is increasingly caused by social noise exposure [2]. At least one million healthy years of life are lost every year from traffic-related environmental noise in western Europe [1]. Soundscape is acoustic environment as perceived or experienced or understood by a person or people, in context [3]. Previous research has concluded that the quality of the soundscape has a significant impact on environmental experience and health [4]. Moreover, experiments have confirmed that exposure to a restorative environment can aid recovery from mental fatigue [5]. Generally, natural landscapes have a greater impact on health than urban landscapes, urban landscapes even have negative impacts on health, whereas natural landscapes contribute to short-term recovery from stress or mental fatigue [6]. Zhang [7] found that the natural environment can induce positive emotions and help in restoring attention, and physiological changes caused by negative emotions; thereby, reducing mental fatigue. Medvedev et al. [8] noticed that following a stress task, larger decreases in heart rate were associated with the least eventful soundscapes. Szeremeta and Zannin reported that multiple acoustic indicators can simultaneously affect people’s perception [9]. Subjective response to sound depends on the listener’s mental, social, and demographic relation to the sound source and its context [10]. The natural soundscape is related to the season in some cases. Due to different seasons and climate, the types of biophonic life and geophony that composed natural soundscape are different. Winter natural soundscape in Yellowstone National Park has been explored to evolving demands for park access and natural soundscape experiences [11]. The temporal variation and spatial relationships of soundscape components to the landscape in winter in south-central Alaska has been quantified and described [12]. Although previous research has shown that natural soundscapes have a recovery effect on human emotions and stress, research into the recovery effect of sound sources should be improved.

Stress Reduction Theory (SRT) was put forward and developed to understand the aesthetic and affective response as well as to express the emotional and physiological reactions to natural environment [13,14]. The concept of a “restorative environment” refers to an environment that is conducive to people recovering from psychological fatigue and negative emotions associated with stress [15]. This concept is based on the Attention Restorative Theory proposed by Kaplan [16,17]. Four basic characteristics of a “restorative environment” are generally considered as being away, fascination, extent, and compatibility [16]. The natural environment contributes to the recovery of stress and attention fatigue in terms of cognition, emotion, and psychology [18]. Moreover, a recent meta-analysis of the positive and negative effects of natural environments exposure has strengthened empirical support for Ulrich’s theory, in that exposure to nature was found to be positively correlated with positive emotions and negatively correlated with negative emotions [19].

The acoustic environment can have negative or positive effects on human health, and natural soundscapes such as birdsong can have a positive impact on recovery [20]. A systematic review has been explored to study association between positive health-related effects and soundscapes perceptual constructs [21]. It is indicated that sound perception can act as an enhancer of the human experience in the urban realm, from a health-related point of view. Natural sounds were being part of a pleasant and “quiet” experience that supported recovery and induced “soft fascination” [22]. Skin conductance level recovery tended to be faster during natural sound than noisy environments, which suggested that nature sounds facilitate recovery from sympathetic activation after a psychological stressor [22]. In a virtual natural environment, parasympathetic activation subjected to sounds of nature, which indicated that enhanced stress recovery may occur in such surroundings [23]. Birdsong is a natural sound source with strong potential for restoration, which can be used to study the restoration effects of birdsong landscapes in urban parks. In [24], seven types of sound that frequently appear in urban parks, including wind, road traffic noise, and bird sounds, were quantified in accordance with human comfort level. The results indicated that bird sounds were considered to be the most harmonious and pleasant sound in various urban parks when compared with artificial sounds and running water sounds [24]. Ratcliffe and Gatersleben [25] determined that birdsong can help people recover quickly from stress, and that bird sounds rated as high in perceived restorative potential were associated with green spaces, spring and summer, daytime, and active behaviors in the environment. However, not all birdsong sounds can promote stress recovery, and it is related to semantic values and restorative perceptions of natural stimuli [26]. Therefore, although natural sound and birdsong soundscapes can promote recovery from stress and negative emotions, the detailed relationship between birdsong soundscape and perceived recovery remains unknown.

Thus, the aim of this study is to evaluate the effects of different types of birdsong on the perceived restorativeness soundscape scale (PRSS) in different seasons and at different heights in an urban park. The influence of demographic and social factors on PRSS evaluations is also determined. The results may be used for the planning and designing of birdsong scenes in urban parks.

## 2. Materials and Methods

Eight research sites were selected in a city park with abundant space composition, plant species, and a large number of visiting. As the highest frequency of occurrences through observations, four common bird types of resident bird as sound sources of birdsong were selected. Soundscape perception and the perceived restorativeness with different types of birdsong were investigated in different seasons and at different heights. The effects and relationships of perceived restorativeness soundscape were then analyzed.

### 2.1. Site and Sound Source Selection

**Figure 1:**
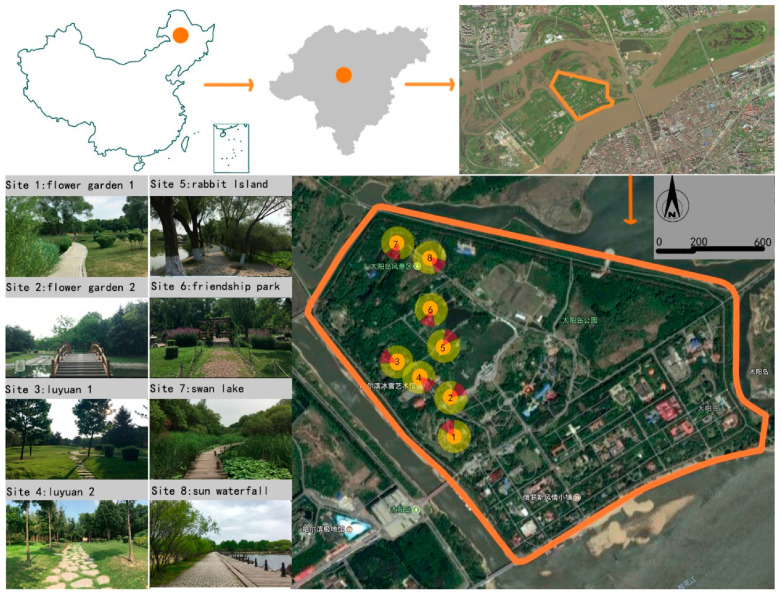
Map and aerial photos of the study area, site selection photographs, and plan view of Harbin Sun Island Park.

Sun Island Park was selected as the urban park for research site in Harbin of China, which is mainly inhabited by native residents, as shown in Figure 1. It is a typical urban park in a typical city with many local residents and a good ecological environment. Compared to other urban parks, the area of Sun Island Park is large with 38 square kilometers, and the plants tend to natural communities. Coniferous trees and deciduous tree species are diverse and rich, and there are rich layers of shrubs and shrubs. There are many tree species, and the combination of trees, shrubs, and grass is rich. The island contains waters, which supply a suitable ecological environment for insects and birds. Plant habitats have an impact on the types and numbers of birds, which also determines the richness of the soundscape of birdsongs. Abundant landscape elements meet the requirements for birds living and soundscape, which can also promote interaction between the visual and acoustic aspects of the perceived restorativeness soundscape.

Eight sites were selected in Sun Island Park. These are the Flower Garden (two sites), Luyuan (two sites), Rabbit Island, China–Japan Friendship Park, Swan Lake, and Sun Waterfall, as shown in Figure 1. 

Space composition and typical plants for those eight sites are shown in Table 1. The sites were all composed of many landscape elements, rich plant habits, and visitors. Most of them are composed by deciduous forests or broad-leaved forests, bushes, or grasslands or/and waters. They all provide good ecological environments and living habitats for types of birds. In terms of space compositions, the sites all have the characteristics of high connectivity, high integration, and easy access, based on the principles of space syntax.

Four types of birds were selected as natural sound sources of birdsongs in those eight sites. This survey would be conducted both in summer and winter; therefore, resident birds would be selected rather than migratory birds, due to cold weather in winter. Through the field survey for the birds in selected sites, fifteen types of resident birds were observed. Four types of resident birds had the most occurrence, which were also best known to people. They are sparrow, woodpecker, magpie, and crow. The frequencies of these four birds are in the range of 1600Hz–8000Hz.

For detecting the effect of each birdsong on perceived restorativeness, sound clips using a high-fidelity recorder of FOSTEX FR-2LE with those four types of birdsongs as the foreground sound were recorded respectively, at the daytime in summer when the birds were singing. For detecting the effect of birdsong on relationships between social factors and perceived restorativeness, Audacity software was used to combine the four birdsongs as a natural environment to construct a comprehensive acoustic environment. At the field surveys, a mini stereo of JBL Flip 4 was used to playback the birdsongs and comprehensive sounds. The stereo was tied to a folding rod and hidden on the shrubs and trees, meeting the requirements of different heights of the sound source during the field investigation. The playback volume was adjusted appropriately based on the selected sites, space types, and environment background sounds, which with excessively high or low volume would affect people’s evaluations.

The sites were selected based on one of the principles of moderate crowds; therefore, it was easy for people to enter the survey sites. The time of the survey was selected in the morning and afternoon of sunny days when the activities were much frequent. At the sites of field survey, respondents filled in the form of demographic and social information firstly as mentioned in Section 2.2. Four birdsongs were played for 30 s respectively. To reduce the impact of the order of birdsong on evaluations, the order of birdsong was played randomly. When the playback of each birdsong ended, the respondents filled in the questionnaires of evaluations, respectively. The combination of birdsongs was played 30 s at the height of 0.5 m, 2 m, and 4 m, respectively. Similarly, when the playback of combination sounds at each height ended, the respondents filled in the questionnaires of evaluations, respectively.

### 2.2. Questionnaire Design

The study was conducted using an on-site questionnaire survey for local respondents. The respondents were people who came to the park spontaneously. After entering the research sites, the visitors were asked whether they could participate in the questionnaire survey. After agreeing, the respondents would perceive the site environment, looked at the landscape component, listen to the sounds and fill in the questionnaires. Based on the demographic and social indicators described in Table 2, the respondents were enquired about their gender, age, education level, occupation, and stress level. A database was established, which included gender (male and female), age (19 or less, 20–29, 30–39, 40–49, 50–59, 60 or more), education level (primary, middle, undergraduate, postgraduate), occupation (design related work, non-design related work), and stress level in the last month (very low, low, moderate, high, very high). These data were used in the subsequent analysis.

The four components of a restorative environment are defined as follows [16]. “Being away” involves a physical or conceptual shift away from the present situation to a different environment, allowing tired cognitive structures to rest and allowing other ones to activate [16,26]. A “Fascinating” stimulus is one that calls forth involuntary attention. It attracts people and prevents them from getting bored, allowing them to function without having to use directed attention [16]. “Compatibility” depends on an individual’s inclinations as much as the environment. A high match between the individual and the environment results in the individual using little directed attention as few differences need to be resolved [26]. “Extent” refers to the coherency, scope, and richness of an environment that enables the individual to feel as if it is a “whole other world”, which has explorative potential [17]. This study employed a previous PRSS, as shown in Table 3, developed for an urban park environment based on general perceived restorativeness scales [26], which includes 13 items: four items of theoretical environmental restoration, three items for “fascination”, four items for “being away”, three items for “compatibility”, and three items for “extent”.

Additionally, evaluations of acoustic comfort, perceived intensity, and preference for the birdsong soundscape in the urban park were also examined in the survey, as shown in Table 4. The respondents were asked to describe the items during summer and winter interview periods to explore seasonal differences in the perceived restorativeness soundscape. The following seven-point scale of agreement was employed: not at all (1), very little (2), a little (3), somewhat (4), a fair bit (5), very much (6), and completely (7). Three birdsong heights of 0.5 m, 2 m, and 4 m were employed to explore the effect of height on the perceived restorativeness soundscape.

### 2.3. Data Analysis

The software SPSS 18.0 (IBM, Armonk, USA) was used to calculate statistical parameters based on the data collected from the surveys. The mean values of soundscape perception for different types of birdsongs in different seasons were analyzed, as well as the PRSS at different heights. Common statistical methods were used in this study. Paired samples tests were used to check significance differences for different heights. Spearman’s correlation analysis was used to find the correlations between age, education level, stress level, and PRSS for different heights. Non-parametric tests were used to explore the effects of gender and occupation on PRSS for different heights. Moreover, factor analysis was used to analyze the relationship between PRSS evaluations and thirteen issues of various indicators.

## 3. Results

A total of 240 valid questionnaires were received during the survey. The respondents were selected randomly. The numbers of male and female respondents were 110 and 130, respectively. Table 5 shows the age distribution. People between 20–39 years old were around 45 % of the respondents. The numbers of other groups of ages were rather similar. In terms of education levels, the number in primary group was 22, middle group was 81, undergraduate group was 95, and postgraduate was 42. The classification of occupation was divided into two groups of design related work, and non-design related work. The numbers were 55 and 185, respectively. In terms of stress level, seven respondents felt very little stress in this survey. Fifty-three and 132 respondents felt a little and moderate stress, respectively, which occupied 77 % of the respondents. Thirty-one respondents felt much stress, and 17 felt stressed very much.

### 3.1. Soundscape and Perceived Recovery of Four Types of Birdsong in Summer and Winter

#### 3.1.1. Evaluation of Soundscape Perception

**Figure 2:**
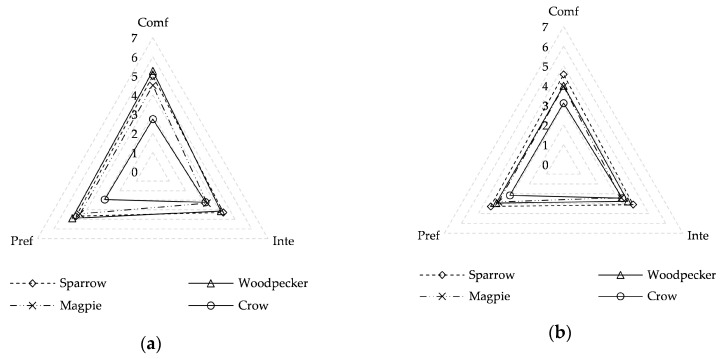
Mean values of sound comfort (Comf), perceived intensity (Inte), and preference (Pref) for different birdsongs in different seasons: (**a**) summer; (**b**) winter.

As shown in Figure 2, four types of birdsong (sparrow, woodpecker, magpie, and crow) were evaluated according to sound comfort, perceived intensity, and preference in both summer and winter. The mean sound comfort values of woodpecker, sparrow, magpie, and crow sounds in summer are 5.26, 4.99, 4.51, and 2.74, respectively, which indicate that the woodpecker sound makes people feel most comfortable in summer. The corresponding mean values in winter are 4.00, 4.57, 3.96, and 3.12, respectively, which show that the sparrow sound makes people feel most comfortable in winter. The different types of birdsong exhibit different sound comforts in different seasons; however, the crow sound exhibits the lowest sound comfort values of all types of birdsong both in summer and winter and the lowest mean sound preference values (2.92 in summer and 3.16 in winter). The perceived intensity of the sparrow sound is the highest, at 4.29 in summer and 4.09 in winter. The sound comfort and preference of birdsong is generally lower in winter than in summer. This is mainly because the air temperature in Harbin is low in winter, which affects subjective evaluations of sound comfort and preference. Another reason may be that a mini stereo as the sound source was used in the field investigation. During the summer survey, the sound source could be well hidden, and the sound could be integrated into the environment; in winter, due to the withering of the trees, the source could not be well hidden. Perhaps the respondents saw the stereo and produced a feeling of unreality. Seasonal differences in the perceived intensity of the four types of birdsong are small.

#### 3.1.2. PRSS Evaluations

**Figure 3:**
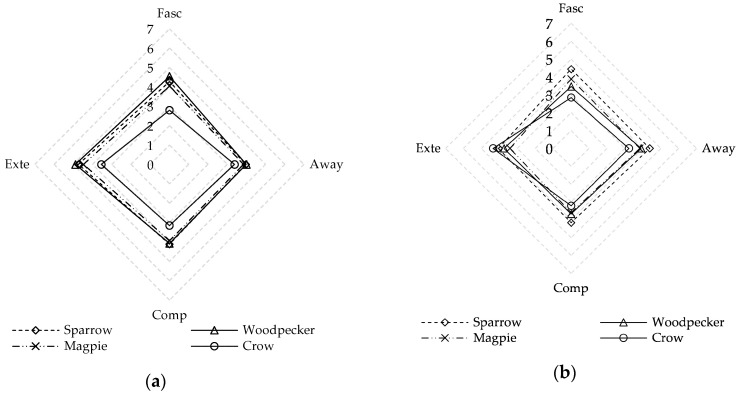
Comparison of perceived restorativeness characteristics for four bird types: (**a**) summer; (**b**) winter. Fasc = fascination; Away = being away; Comp = compatibility, and Exte = extent.

As shown in Figure 3, the four types of birdsong were also evaluated according to the PRSS items of fascination, being away, compatibility, and extent in summer and winter. In summer, the mean values of crow sounds (3.22) are the lowest of all types of birdsong: 2.80 for fascination, 3.38 for being away, 3.16 for compatibility, and 3.35 for extent. Crow sounds also receive the lowest evaluation in winter (3.41). Woodpecker sounds have the highest mean values of all birdsong evaluations (4.38): 4.55 for fascination, 4.00 for being away, 4.08 for compatibility, and 4.89 for extent in summer. However, sparrow sounds (4.26) have the highest evaluations in winter. Thus, different types of birdsong have different perceived restorative potential.

Table 6 presents the correlations between the evaluations of soundscape perception and the four PRSS items for the four bird sounds in summer and winter. Both sound comfort and preference are positively associated with PRSS for all four types of birdsongs in both seasons. Perceived intensity has no significant correlation with PRSS (*p* > 0.05).

In summer, the PRSS items fascination, being away, and compatibility are typically positively correlated with soundscape perception. However, there is no significant correlation between compatibility and the perceived soundscape (*p* > 0.05) for sparrow birdsong. Sparrows are so common in city, residential area, campus, and everywhere in daily life that people are familiar with them. In spaces with more natural environments, people expect to hear much different natural sounds. The perceived intensity of crow birdsong is negatively correlated with fascination (*p* < 0.05), which indicates that the higher the perceived intensity, the lower the fascination. In winter, fascination is typically significantly associated with sound comfort and preference (*p* < 0.01). Being away is not associated with indicators of soundscape perception for any types of birdsong (*p* > 0.05) except magpie (*p* < 0.01). Specifically, magpie birdsong exhibits the greatest correlation between soundscape perception and PRSS items (*p* < 0.01).

### 3.2. PRSS Evaluations at Different Heights

**Figure 4:**
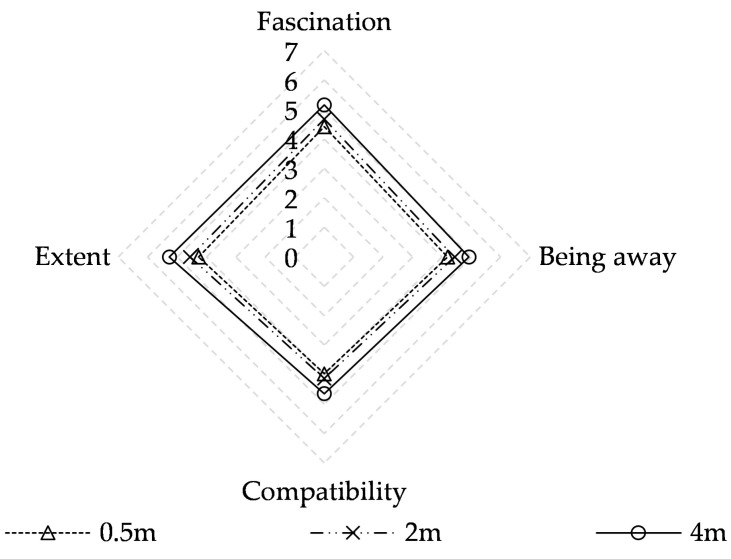
Mean PRSS evaluation scores at different heights.

The mean values of the four PRSS items are shown in Figure 4; the PRSS evaluation scores are 4.21 at 0.5 m, 4.45 at 2 m, and 4.99 at 4 m. Significant differences (*p* < 0.01) are observed in birdsong in the perceived restorativeness soundscape at heights of 0.5 m, 2 m, and 4 m, with the best evaluation at a height of 4 m (Table 7).

Paired samples tests of four components of PRSS namely fascination, being away, compatibility, and extent for perceived restorativeness birdsong at different heights are shown in Table 7. There are significant differences (*p* < 0.01) in the four items at 0.5 m, 2 m, and 4 m, indicating that the evaluation scores increase with increasing birdsong height for all items. Notably, each component of perceived restorativeness at the height of 4 m is better than evaluations at other heights. This is likely because, at that height, birdsong is integrated into the background rather than the foreground, creating a compatible audio-visual environment. Another reason is mainly that people generally thought that the birdsongs should be at a high place, and this idea made people think the high place for birdsong was comfortable and appropriate.

As shown in Figure 4, 5.27 is the highest score for the indicator of extent and 4.64 is the slowest for compatibility when the birdsong is at 4 m; however, minimal difference is observed in the mean scores among the four items. The perceived restorativeness of birdsong is balanced between fascination, being away, compatibility, and extent. This is also true at 0.5 m and 2 m. These results are similar to a previous study [27], which showed that a landscape containing natural water and high plant coverage matches the visual association of a bird singing; thus, adding birdsong to this landscape will lead to higher restorative potential.

Factor analysis was used to analyze the relationship between PRSS evaluations and thirteen issues of various indicators at the height of 4 m mentioned in 2.2. Varimax rotated principal component analysis was employed to extract the orthogonal factors from 13 indicators. With the criterion factor of eigenvalue > 1, factors are determined as shown in Table 8. Three common factors were extracted, the KMO result was 0.888, and the cumulative contribution rate was 55.6%. Factor 1 (26.3%) is mainly associated with extent, compatibility, and being-away. Factor 2 (20.6%) is generally associated with fascination. Factor 3 (8.7%) is with sounds hinder. It is interesting to note that being-away, compatibility, and extent are much more important than fascination for PRSS evaluations when the birdsongs were at the height of 4 m in these survey sites. People felt more relaxed than immersed in birdsongs.

### 3.3. Correlations Between Demographic/Social Factors and Perceived Restorativeness at Different Heights

Table 9 presents the relationships between the PRSS at different heights and several social/demographical factors. No significant difference is observed between genders (*p* > 0.05) or occupations (*p* > 0.05). Age is positively associated with PRSS (*p* < 0.01) at heights of 0.5 m and 2 m. Education is negatively correlated with PRSS (*p* < 0.05) at heights of 0.5 m and 4 m. These results are similar to previous studies [28,29], which illustrate that age and education typically affect sound preferences, and age is positively correlated with pleasantness in urban open public spaces. Stress level is negatively associated with PRSS evaluation scores (*p* < 0.01) at a height of 0.5 m, indicating that people with less stress over the last month give higher PRSS evaluation scores.

Correlations between the four PRSS items and social/demographical factors at different heights are also shown in Table 9. All four items are typically associated with age at heights of 0.5 m and 2 m, with the exception of compatibility at 0.5 m. Education is negatively correlated with compatibility at 0.5 m (*p* < 0.01), 2 m *(p* < 0.05), and 4 m *(p* < 0.01). Stress level is negatively associated with being away (*p* < 0.01), compatibility (*p* < 0.01), and extent (*p* < 0.01) at a height of 0.5 m, and negatively associated with compatibility (*p* < 0.05) and extent (*p* < 0.01) at 2 m. Thus, age, education, and stress level are significantly associated with most PRSS items at 0.5 m and 2 m, respectively. There are no significant correlations between PRSS items and demographic/social factors at 2 m.

## 4. Discussions

The results indicate four main findings. First, the type of birdsong influences PRSS evaluations. Compared to sparrows, woodpeckers, and magpies, crows score the lowest value for birdsong PRSS both in summer and winter. Woodpeckers and sparrows score the highest values in summer and winter, respectively, indicating greater sound comfort and preference. This is similar to the results of previous studies [25,30], which showed that perceptions of restorative value vary between bird species, and that high perceived restorative potential depends on green spaces, seasons, and behaviors in the environment. They are both positively associated with PRSS, and not associated with perceived sound intensity. Thus, the addition of comfortable or favored birdsong will likely improve the perceived restorativeness, especially in urban parks with a constructed natural element. That is, creating a suitable habitat for birds and introducing multiple species of birds will improve the perceived restorativeness of sites.

Second, the perceived restorativeness of the soundscape differs with the season. In summer, the PRSS for birdsong is significantly correlated with sound comfort and preference, especially the components of fascination and being away. In winter, the PRSS is also significantly correlated with sound comfort and preference, most notably the component of fascination. Thus, in terms of PRSS, fascination is an important factor for sound comfort and preference. The study area of Harbin has very cold winters, with an average temperature of 19 °C below zero, in which ice and snow landscape enrich the space environment; therefore, interactions between temperature, vision, and acoustics are thought to influence the evaluation of soundscapes and perceived restorativeness. The results are similar to those of the soundscape framework [31], which indicates that the physical environment is associated with subjective evaluation. Since a good audio-visual environment can influence perceived restorativeness, the construction of rich ice and snow landscapes may have an effect on perception recovery. Moreover, the congruence or coherence between sound and image influences preferences. Coherent combinations are rated higher than the mean of the component stimuli [32]. In the future works, more research on ice and snow landscapes and winter soundscapes can be conducted. It could be conducted in virtual reality urban park with different seasons and scenes to research the effect of winter landscape and soundscape, which showed the potential importance as a tool [23]. Additionally, sounds from speakers sometimes were not really natural in some cases. It is depended on environment, distance, visual, climate and perception, which are related to the concept of sound effects and urban design [33].

Third, the PRSS differs at different heights, with the highest evaluation scores at a height of 4 m. It is mainly because that low birdsong heights are close to people so become part of the foreground sound, leading to a low subjective evaluation of comfort. Conversely, a height of 4 m is located among the trees, which suggests consistency between birdsong and tall trees. The visual landscape of the green environment and the soundscape of natural sources are integrated at this height. This means that the interaction between the audio-visual environment is consistent and positive, and the effect on evaluations and perceived restorativeness is positive. Moreover, in this study, the real birdsong was instead of a stereo hidden in the shrub. People heard the birdsongs but did not see the real birds at low height. This is another likely reason for the low subjective evaluation of comfort. If a real bird were close, this would most likely be a very positive experience (at least as an overall experience including all senses), likely to stir “soft fascination” [16]. However, it is not only the effect of soundscape on perceived restorativeness, but also on the visual, behavioral, psychological, and environment. Its salient informational content or due to the drastic impact of the loss of sound quality on observer appreciation, for example, in urban green spaces, natural spaces and cultural landscapes [32]. Additionally, based on the method of factor analysis, three common factors were extracted for PRSS evaluations when the birdsongs were at the height of 4 m in these survey sites. The result is similar with the production of a perceived restorativeness soundscape scale for urban park in UK, which showed the PRSS is appropriate to these sites in Harbin of China [26].

Fourth, demographic and social factors impact PRSS evaluations. At heights of 0.5 m and 2 m, the older the respondent, the better the PRSS evaluation. This is because the elderly’s auditory perception is insensitive. When the birdsong is at a relatively low height as the foreground sound, the older respondents’ perception and favor of the birdsong is just appropriate. In contrast, the birdsong may be relatively noisy for young people at these heights. Education and stress levels over the last month are negatively associated with PRSS, especially at a height of 0.5 m. Therefore, appropriate activities should be provided in city parks to ensure a perception recovery environment, especially for subjects with high levels of education and stress.

Additionally, the potential influence of using speaker would be conducted in the future work. In this study, the types of space were kinds of open or close or semi-open-close, and the natural components were rich. The volume of birdsongs was adjusted to integrate in the original environment as much as possible. Excessively high or low volume would affect people’s evaluations; however, the details of volume effects and spectrum characteristics of different birds on perceived restorativeness and soundscape perception need further studies. Moreover, the height of speaker installation and hidden or not also need to be explored in urban parks.

## 5. Conclusions

The effects of four birdsong types and heights on the perceived restorativeness soundscape scale were explored both in summer and winter in Sun Island Park as a city park in China. Demographic and social factors were also examined to impact the perceived restorativeness soundscape scale. Eight sites were selected, and 240 respondents took part in the field investigation. They have suggestions for urban park planning and design, especially with constructed natural elements. Creating a suitable habitat for multiple species of birds will improve perceived restorativeness. Interactions and correlations between temperature, visions of the natural landscape, and acoustics in nature all improve evaluations of the soundscape and perceived restorativeness. Further research on ice and snow landscapes and winter soundscapes can be conducted. More studies of effects of PRSS on species of birds in different seasons, combing landscape, climate, spaces, and functions, would be explored in city parks. Moreover, effect of speaker installations hidden with natural sounds in high trees would be detected in urban park.

## Figures and Tables

**Table 1:** Space composition and typical plants for eight sites in Sun Island Park.

Site Number	Site Name	Space Composition	Typical Plants
1	Flower Garden 1	Shrub and woodland (open)	*Maidenhair, coleus, prunus triloba, syinga microphylla, saliz matsudana, embla, poplar*
2	Flower Garden 2	Hard paving, grass, and shrub	*Begonia semperflorens, zinnia, sabina procumbent, Mongolian oak, saliz matsudana*
3	Luyuan 1	Arbor and woodland (open)	*Mongolian oak, Prunus maackii*
4	Luyuan 2	Arbor and woodland	*Zinnia, clove, embla, prunus triloba, petunia, day lily, Border Privet, Populus albaxp*
5	Rabbit Island	Arbor and woodland	*Saliz matsudana, prunus triloba*
6	China–Japan Friendship Park	Arbor, shrub, and woodland	*Clove, Saliz matsudana, embla, Border Privet, Typha*
7	Swan Lake	Wetland	*Saliz matsudana, Typha, Syringa reticulata*
8	Sun Waterfall	Hard paving and arbor	*Mongolian oak, clove, Saliz matsudana, embla*

**Table 2:** Demographic and social data determined by the questionnaire.

Demographic and Social Indicators	Categorization and Scale
Gender	1: Male; 2: Female
Age	1: 19 or less; 2: 20–29; 3: 30–39; 4: 40–49; 5: 50–59; 6: 60 or more
Education level	1: Primary; 2: Middle; 3: Undergraduate; 4: Postgraduate
Occupation	1: Design related work; 2. Non-design related work
Stress level in last month	1: Very little; 2: A little; 3: Moderate; 4: Much; 5: Very much

**Table 3:** Perceived restorativeness soundscape scale items.

Components	No.	Questions
Fascination	F-1	I find this acoustic environment appealing.
	F-2	In this place my attention is drawn by many interesting sounds.
	F-3	I am engrossed in this acoustic environment.
Being away	B-4	When I hear these sounds, I can do something different than usual.
	B-5	This acoustic environment is different to what I usually hear.
	B-6	This acoustic environment is a refuge from unwanted distractions.
	B-7	I feel free from routine and responsibility in this acoustic environment.
Compatibility	C-8	This acoustic environment fits with my preference.
	C-9	I can quickly get used to this type of acoustic environment.
	C-10	Hearing these sounds hinders what I want to do here.
Extent	E-11	All the sounds I am hearing belong here.
	E-12	All the sounds merge to form a coherent sonic environment.
	E-13	The acoustic environment suggests that the size of this place is limitless.

**Table 4:** Factors of soundscape perception evaluations and scores from 1 to 7.

Factors	Score (1–7)						
Acoustic comfort	1	2	3	4	5	6	7
Perceived intensity	1	2	3	4	5	6	7
Preference	1	2	3	4	5	6	7

**Table 5:** Number of respondents for each age group of the survey.

Age Group (Years)	Frequency (*n*)	Percentage
<19	34	14.2
20–29	62	25.8
30–39	47	19.6
40–49	31	12.9
50–59	37	15.4
>59	29	12.1
Total	240	100

**Table 6:** Spearman’s Rho correlations between soundscape perception evaluations and the perceived restorativeness soundscape scale (PRSS) for different birdsongs. Fasc = fascination; Away = being away; Comp = compatibility; Exte = extent; Comf = sound comfort; Inte = perceived intensity; and Pref = preference.

**Summer**	**Sparrow**			**Woodpecker**		
	**Comf**	**Inte**	**Pref**	**Comf**	**Inte**	**Pref**
PRSS	0.633 **	0.288	0.356 *	0.624 **	0.226	0.645 **
Fasc	0.696 **	0.259	0.515 **	0.569	0.264	0.605 **
Away	0.447 *	0.454 **	0.285	0.395 *	0.437 *	0.608 **
Comp	0.349	0.111	0.153	0.603 **	0.084	0.562 **
Exte	0.305	0.079	−0.027	0.142	−0.101	0.304
Summer	Magpie			Crow		
	Comf	Inte	Pref	Comf	Inte	Pref
PRSS	0.546 **	0.342	0.531 **	0.538 **	0.305	0.681 **
Fasc	0.616 **	0.348	0.63 **	0.676 **	−0.413 *	0.754 **
Away	0.478 **	0.438 *	0.488 **	0.561 **	0.409 *	0.575 **
Comp	0.51 **	0.29	0.559 **	0.403 *	0.241	0.424 *
Exte	0.151	0.049	0.076	0.287	0.413 *	0.216
Winter	Sparrow			Woodpecker		
	Comf	Inte	Pref	Comf	Inte	Pref
PRSS	0.59 **	0.323	0.551 **	0.436 **	0.081	0.462 **
Fasc	0.656 **	0.218	0.636 **	0.323 *	0.077	0.723 **
Away	0.264	0.207	0.167	0.009	0.143	0.152
Comp	0.18	0.025	0.226	0.414 *	0.096	0.386 *
Exte	0.245	0.413 *	0.313	0.079	-0.085	0.039
Winter	Magpie			Crow		
	Comf	Inte	Pref	Comf	Inte	Pref
PRSS	0.602 **	0.336	0.765 **	0.469 **	0.321	0.546 **
Fasc	0.572 **	0.308	0.796 **	0.555 **	−0.414 *	0.664 **
Away	0.523 **	0.314	0.745 **	0.298	0.171	0.213
Comp	0.51 **	0.295	0.743 **	0.146	0.289	0.318
Exte	0.308	0.337	0.259	0.321	0.215	0.355 *

** Significant at *p* < 0.01; * Significant at *p* < 0.05.

**Table 7:** Paired samples test results of PRSS and PRSS item evaluations at different heights. Fasc = fascination; Away = being away; Comp = compatibility; and Exte = extent.

Indicators	Mean	Std. Deviation	Sig. (2-Tailed)
PRSS (4 m)–PRSS (0.5 m)	0.79	1.12 **	0.000
PRSS (4 m)–PRSS (2 m)	0.55	0.91 **	0.000
PRSS (2 m)–PRSS (0.5 m)	0.24	0.71 **	0.000
Fasc (4 m)–Fasc (0.5 m)	0.74	1.46 **	0.000
Fasc (4 m)–Fasc (2 m)	0.48	1.28 **	0.000
Fasc (2 m)–Fasc (0.5 m)	0.26	1.12 **	0.000
Away (4 m)–Away (0.5 m)	0.73	1.17 **	0.000
Away (4 m)–Away (2 m)	0.49	1.00 **	0.000
Away (2 m)–Away (0.5 m)	0.24	0.84 **	0.000
Comp (4 m)–Comp (0.5 m)	0.68	1.29 **	0.000
Comp (4 m)–Comp (2 m)	0.51	1.14 **	0.000
Comp (2 m)–Comp (0.5 m)	0.16	0.85 **	0.004
Exte (4 m)–Exte (0.5 m)	1.00	1.50 **	0.000
Exte (4 m)–Exte (2 m)	0.70	1.15 **	0.000
Exte (2 m)–Exte (0.5 m)	0.30	1.08 **	0.000

** Significant at *p* < 0.01.

**Table 8:** Factor analysis of PRSS issues.

PRSS with Kaiser–Meyer–Olkin Measure of Sampling Adequacy: 0.888; Cumulative: 55.6 %.			
PRSS Issues	1 (26.3 %)	2 (20.6 %)	3 (8.7 %)
C9	0.728	0.187	−0.191
E13	0.709	−0.008	0.170
E12	0.669	0.235	0.041
C8	0.660	0.405	−0.222
B7	0.643	0.371	−0.189
B6	0.624	0.238	0.101
B5	0.508	0.295	−0.008
E11	0.483	0.312	0.372
F2	0.177	0.765	0.019
F3	0.299	0.746	−0.031
F1	0.258	0.714	0.046
B4	0.141	0.625	0.125
C10	−0.061	0.064	0.901

**Table 9:** Non-parametric tests and Spearman’s Rho correlations between PRSS and demographic/social factors at different heights.

Indicators	Gender	Age	Education	Occupation	Stress Level
PRSS—0.5 m	−0.020	0.175 **	−0.153 *	0.051	−0.231 **
PRSS—2 m	−0.016	0.227 **	−0.104	0.028	0.106
PRSS—4 m	−0.052	0.045	−0.147 *	0.058	0.028
Fascination—0.5 m	0.015	0.170 **	−0.097	0.024	−0.088
Being away—0.5 m	−0.027	0.132 *	−0.120	0.039	−0.248 **
Compatibility—0.5 m	−0.003	0.124	−0.222 **	0.080	−0.185 **
Extent—0.5 m	−0.037	0.175 **	−0.093	0.004	−0.240 **
Fascination—2 m	0.029	0.205 **	0.004	−0.011	0.065
Being away—2 m	−0.026	0.150 *	−0.114	0.049	−0.119
Compatibility—2 m	−0.007	0.212 **	−0.151 *	0.048	−0.142 *
Extent—2 m	−0.063	0.165 *	−0.168 **	0.012	−0.176 **
Fascination—4 m	−0.068	0.014	−0.069	−0.038	0.091
Being away—4 m	−0.076	−0.038	−0.118	0.072	−0.028
Compatibility—4 m	0.018	0.028	−0.189 **	0.099	−0.048
Extent—4 m	−0.086	0.074	−0.121	0.025	−0.058

** Significant at *p* < 0.01; * significant at *p* < 0.05.

## References

[1] World Health Organization (2018). Environmental noise guidelines for the European Region.

[2] Basner M., Babisch W., Davis A., Brink M., Clark C., Janssen S., Stansfeld S. (2014). Auditory and non-auditory effects of noise on health. Lancet.

[3] International Standardization Organization (2014). ISO 12913-1:2014—Acoustics—Soundscape—Part 1: Definition and Conceptual Framework.

[4] He M., Pang H. (2016). A review of soundscape research history and progress. Landsc. Archit..

[5] Berto R. (2005). Exposure to restorative environments helps restore attentional capacity. J. Environ. Psychol..

[6] Velarde M., Fry G., Tveit M. (2007). Health effects of viewing landscapes—Landscape types in environmental psychology. Urban For. Urban Green..

[7] Zhang Y. (2014). Research on soundscape restorative benefits of urban open space and promotion strategy of the acoustic environment quality. New Archit..

[8] Medvedev O., Shepherd D., Hautus M. (2015). The restorative potential of soundscapes: A physiological investigation. Appl. Acoust..

[9] Szeremeta B., Zannin P.H.T. (2009). Analysis and evaluation of soundscapes in public parks through interviews and measurement of noise. Sci. Total. Environ..

[10] Ren X., Kang J., Zhu P., Wang S. (2018). Soundscape expectations of rural tourism: A comparison between Chinese and English potential tourists. J. Acoust. Soc. Am..

[11] Saxen S.W. (2008). Park visitors and the natural soundscape: Winter experience dimensions in Yellowstone National Park. Ph.D. Thesis.

[12] Mullet T.C., Gage S.H., Morton J.M., Huettmann F. (2015). Temporal and spatial variation of a winter soundscape in south-central Alaska. Landsc. Ecol..

[13] Ulrich R.S. (1983). Aesthetic and Affective Response to Natural Environment. Behavior and the Natural Environment.

[14] Ulrich R.S., Simons R.F., Losito B.D., Fiorito E., Miles M.A., Zelson M. (1991). Stress recovery during exposure to natural and urban environments. J. Environ. Psychol..

[15] Kaplan S. (1983). A Model of Person-Environment Compatibility. Environ. Behav..

[16] Kaplan R., Kaplan S. (1989). The Experience of Nature: A Psychological Perspective.

[17] Kaplan S. (1995). The restorative benefits of nature: Toward an integrative framework. J. Environ. Psychol..

[18] Jeon J.Y., Hong J.Y. (2015). Classification of urban park soundscapes through perceptions of the acoustical environments. Landsc. Urban Plan..

[19] Menardo E., Brondino M., Hall R., Pasini M. (2019). Restorativeness in Natural and Urban Environments: A Meta-Analysis. Psychol. Rep..

[20] Aletta F., Oberman T., Kang J. (2018). Associations between Positive Health-Related Effects and Soundscapes Perceptual Constructs: A Systematic Review. Int. J. Environ. Res. Public Heal..

[21] Alvarsson J.J., Wiens S., Nilsson M. (2010). Stress Recovery during Exposure to Nature Sound and Environmental Noise. Int. J. Environ. Res. Public Heal..

[22] Cerwén G., Pedersen E., Pálsdóttir A.M. (2016). The Role of Soundscape in Nature-Based Rehabilitation: A Patient Perspective. Int. J. Environ. Res. Public Heal..

[23] Annerstedt M., Jönsson P., Wallergård M., Johansson G., Karlson B., Grahn P., Hansen Å.M., Währborg P. (2013). Inducing physiological stress recovery with sounds of nature in a virtual reality forest—Results from a pilot study. Physiol. Behav..

[24] Ma H., Wang D. (2012). Primary quantification of soundscape elements in urban parks. Noise Vib. Control.

[25] Ratcliffe E., Gatersleben B., Sowden P.T. (2016). Associations with bird sounds: How do they relate to perceived restorative potential?. J. Environ. Psychol..

[26] Payne S.R. (2013). The production of a Perceived Restorativeness Soundscape Scale. Appl. Acoust..

[27] Zhao J., Xu W., Ye L. (2018). Effects of auditory-visual combinations on perceived restorative potential of urban green space. Appl. Acoust..

[28] Yu L., Kang J. (2010). Factors influencing the sound preference in urban open spaces. Appl. Acoust..

[29] Deng L., Kang J., Zhao W., Jambrosic K. (2020). Cross-National Comparison of Soundscape in Urban Public Open Spaces between China and Croatia. Appl. Sci..

[30] Ratcliffe E., Gatersleben B., Sowden P.T. (2013). Bird sounds and their contributions to perceived attention restoration and stress recovery. J. Environ. Psychol..

[31] Kang J. (2006). Urban Sound Environment.

[32] Carles J.L., Barrio I.L., De Lucio J.V. (1999). Sound influence on landscape values. Landsc. Urban Plan..

[33] Cerwén G. (2018). On the Intersection Between Speaker Installations and Urban Environments. Multi-Objective Optimization of Industrial Power Generation Systems.

